# Fenotipo de envejecimiento saludable de personas mayores en Manizales

**DOI:** 10.7705/biomedica.4799

**Published:** 2020-03-30

**Authors:** Carmen Lucía Curcio, Andrés Fernando Giraldo, Fernando Gómez

**Affiliations:** 1 Programa de Investigaciones en Gerontología y Geriatría, Facultad de Ciencias para la Salud, Universidad de Caldas, Manizales, Colombia Universidad de Caldas Facultad de Ciencias para la Salud Universidad de Caldas Manizales Colombia

**Keywords:** envejecimiento saludable, fenotipo, biomarcadores, determinantes sociales de la salud, envejecimiento, Healthy aging, phenotype, biomarkers, social determinants of health, aging

## Abstract

**Introducción.:**

El fenotipo de envejecimiento saludable está presente en aquellos individuos que envejecen con poca morbilidad, sin deterioro funcional ni cognitivo, y con un nivel aceptable de bienestar y de participación social.

**Objetivo.:**

Establecer la frecuencia del fenotipo de envejecimiento saludable según un modelo multidimensional, uno biomédico y uno psicosocial, y determinar los factores de predicción en cada uno de ellos.

**Materiales y métodos.:**

Se hizo un estudio de diseño transversal, observacional y descriptivo, que incluyó a 402 personas (50,1 % mujeres) de 65 años y más (promedio de edad, 69,2) en el área urbana de Manizales. El fenotipo de envejecimiento saludable se caracterizó en cinco dimensiones: salud metabólica y fisiológica, función física, función cognitiva, bienestar psicológico y bienestar social. Los factores asociados incluyeron aspectos sociodemográficos y de salud.

**Resultados.:**

La prevalencia de envejecimiento saludable fue de 15,5 % en el modelo multidimensional, de 12,3 % en el biomédico y de 63,3 % en el psicosocial. El tener autopercepción de buena salud fue un factor de predicción independiente de envejecimiento saludable en los tres modelos, así como la satisfacción con los ingresos económicos en el modelo biomédico y en el psicosocial. Un tercer factor de predicción fue el estar casado, aunque fue significativo solamente en el modelo psicosocial.

**Conclusiones.:**

La prevalencia del fenotipo de envejecimiento saludable fue baja en el modelo biológico y en el multidimensional (1 de cada 10 personas), y mayor en el modelo psicosocial (6 de cada 10). A pesar de ello, los factores predictores independientes fueron los mismos: la autopercepción de buena o muy buena salud, la satisfacción con los ingresos económicos y el estar casado.

## Abstract (en)

**Introduction:**

The healthy aging phenotype is present in those individuals that age with low morbidity, no functional or cognitive deterioration, and retain an acceptable level of wellness and social participation.

**Objective:**

To establish the frequency of the healthy aging phenotype in older people in the community using a multidimensional, a biomedical, and a psychosocial model and to identify the predicting factors in each model.

**Materials and methods:**

We conducted a cross-sectional, observational and descriptive study. We assessed individuals (n= 402; 50.1% female) aged 65 years or older (69.2 years average) from the urban area of Manizales. The healthy aging phenotype included five domains: biomarkers of physiological and metabolic health, physical capability, cognitive function, and social and psychological wellbeing. We also analyzed sociodemographic- and health-related factors.

**Results:**

In the multidimensional model the prevalence of the healthy aging phenotype was 15.5% while in the biomedical model it was 12.3% and in the psychosocial one it was 63.3%. Good self-perceived health was an independent predictor of healthy aging in all the models assessed. Having enough income was a predictor in the biomedical and psychosocial models while being married was the only significant predictor in the psychosocial model.

**Conclusions:**

The prevalence of the healthy aging phenotype was low in the biological and multidimensional models (1 of every 10 individuals) and higher in the psychosocial one (6 of every 10 persons). However, independent predictor factors were the same in all models: Self-perceived good or very good health, having enough income and being married.

El incremento sostenido del número de personas mayores durante las últimas décadas es evidente en todo el mundo ^1^. Con más de 45 millones de habitantes, Colombia vive actualmente una rápida transformación demográfica y la proporción de personas mayores de 60 años habrá aumentado del 10 al 20 % en el 2050 ^2^.

En su informe sobre envejecimiento y salud ^1^, la Organización Mundial de la Salud (OMS) plantea la necesidad de un cambio de paradigma en la salud de las personas mayores. Se insiste en que la presencia de enfermedades dice poco sobre el impacto que pueden tener en la vida de una persona mayor ^3^. Es claro que, a medida que la población global ha envejecido, se ha fomentado el cambio hacia un discurso positivo sobre el envejecimiento y se ha acuñado el concepto de 'envejecimiento saludable'.

Así, la vejez ya no se presenta como un período inevitable de declive en el que las enfermedades obligan a retirarse de la vida activa ^4^. La visión positiva del envejecimiento ha adoptado numerosas denominaciones: envejecimiento exitoso, óptimo, saludable o positivo, entre otras ^5^^-^^7^, expresiones que algunos autores utilizan indistintamente en los artículos de revisión ^8^^-^^10^. Otros intentan diferenciar entre el envejecimiento saludable, el activo o exitoso y el envejecimiento productivo ^6^^,^^11^^,^^12^. Según Petretto, *et al.*^13^, la tradición europea privilegia el envejecimiento activo y saludable, en tanto que la estadounidense habla de envejecer con éxito.

En una de las primeras definiciones de envejecimiento saludable, este se describe como un proceso complejo de adaptación a los cambios físicos, sociales y psicológicos a lo largo de la vida ^14^, pero en la literatura especializada, el concepto no es constante y no hay consenso sobre su significado, lo que dificulta la medición y el resultado de los programas que lo promueven. Sin embargo, a pesar de las controversias surgidas con respecto a su definición, hay consenso en que el envejecimiento saludable es un resultado positivo en la salud, puesto que las personas conservan la capacidad de funcionar bien y adaptarse a los desafíos del ambiente para su capacidad física, sus funciones fisiológicas, cognitivas y del sistema inmunitario ^15^.

Se ha planteado la existencia de un 'fenotipo de envejecimiento saludable', caracterizado por un mayor grado de complejidad fisiológica en los aspectos de funcionamiento tales como la variabilidad de la frecuencia cardíaca, la estructura neuronal y la arquitectura ósea, que se asocian con un cuerpo biológicamente más joven ^7^^,^^16^. Este fenotipo representa la reserva óptima y la resiliencia biológica para responder y adaptarse a factores ambientales estresantes, lo que se traduce en la ausencia de algunas condiciones médicas (por ejemplo, las enfermedades cardiovasculares, la demencia o el cáncer) y la presencia o el mantenimiento de otros aspectos importantes del funcionamiento humano (por ejemplo, la función física). El fenotipo de envejecimiento saludable es multidimensional, depende de la edad y del sexo, y está determinado por la interacción entre los genes, la impronta epigenética y los factores ambientales ^16^. Además, está conformado por las siguientes dimensiones: salud fisiológica y metabólica, capacidad física, función cognitiva, bienestar social y bienestar psicológico ^7^.

En términos generales, la mayoría de los estudios en geriatría se basan en el conocimiento de la patogenia y el tratamiento de enfermedades específicas. En contraposición, hay una naciente visión del proceso de envejecimiento, denominada "gerociencia", la cual pretende, por medio de una mayor comprensión de los mecanismos propios del proceso de envejecimiento, generar las condiciones para la implementación de medidas tendientes a promover la longevidad saludable, con unos objetivos primordialmente preventivos frente a los meramente terapéuticos ^17^. Un análisis centrado en el nuevo paradigma de salud y el enfoque multidimensional de un envejecimiento saludable, deben ser punto de partida para las políticas e intervenciones que lo promuevan. El conocimiento de los indicadores, la prevalencia y los factores asociados al fenotipo de envejecimiento saludable, tiene importantes implicaciones en cuanto a la forma de abordarlo y estimularlo en la población que envejece.

En la revisión de la literatura que se hizo sobre el tema, no se encontraron estudios en nuestro medio enfocados en la descripción de este fenotipo y de sus principales biomarcadores. En el presente estudio, se tomó el modelo multidimensional propuesto por Lara, *et al.*^18^, el cual incluye la salud fisiológica y metabólica, la capacidad física, y los aspectos psicológicos y sociales abordados desde la perspectiva del bienestar. Utilizando los datos de un estudio prospectivo longitudinal de personas de 65 años y más en Manizales, se buscó establecer la frecuencia del fenotipo de envejecimiento saludable con base en tres modelos, y determinar los factores de predicción en cada uno de ellos, con el fin de fomentar una visión positiva del proceso de envejecimiento, establecer los indicadores más importantes que se deben tener en cuenta en la atención primaria en salud para detectar a aquellas personas con mayores probabilidades de tener un envejecimiento saludable, así como las estrategias para mantenerlos saludables tanto tiempo como sea posible. Solo mediante el establecimiento de los factores determinantes que influyen en el envejecimiento, será posible el desarrollo de estrategias que permitan promover la forma saludable de envejecer.

## Materiales y métodos

### Diseño

Se hizo un estudio transversal, observacional y descriptivo.

### Población y muestra

Se tomaron los datos del *International Mobility in Aging Study* (IMIAS), una investigación prospectiva longitudinal en adultos mayores entre los 65 y los 74 años llevada a cabo entre el 2012 y el 2016 en cinco ciudades del mundo, entre ellas, Manizales ^19^.

Se hizo un muestreo aleatorio por conglomerados (sectores, secciones, manzanas y viviendas). Con un nivel de confianza del 95 %, se estableció un tamaño muestral de 384 personas; además, se incluyeron 27 hombres más para equilibrar las unidades de muestra por sexo. La muestra total fue de 407 personas (203 hombres y 204 mujeres). Para efectos del presente análisis, se tomaron los datos del 2012.

Los criterios de inclusión fueron una edad entre los 65 y los 74 años, no tener deterioro cognitivo según la respuesta a las preguntas de orientación de la prueba cognitiva de Leganés ^20^, y aceptar y firmar el consentimiento informado.

### Indicadores de envejecimiento saludable

La selección de los indicadores se basó inicialmente en la definición propuesta por Lara, *et al.*^18^, complementada con adaptaciones surgidas de las sugerencias de estudios más recientes ^7^^,^^21^^-^^23^. El fenotipo de envejecimiento saludable incluyó cinco dimensiones: salud metabólica y fisiológica, función física, función cognitiva, bienestar psicológico y bienestar social. Se consideró que las personas que cumplían con los cinco criterios tenían envejecimiento saludable. Los indicadores adoptados para cada una de las dimensiones, se presentan en el cuadro 1.

**Cuadro 1:** Dimensiones e indicadores de envejecimiento saludable

Dimensión	Indicadores
Salud fisiológica y metabólica	Inflamación evaluada según los niveles de proteína C reactiva ultrasensible (ultra-sensitive CRP enzyme-linked método de inmunoturbidimetría CRP-L3 de Roche^®^. Se dividió en baja (<1 mg/L) versus el resto (24).
	Metabolismo de la glucosa medido con la prueba de la hemoglobina ‘glicosilada’ (HbA1c); se consideró normal si era <5,7 (25).
Función física	Fuerza de agarre medida con un dinamómetro (Jamar ulic Hand Dynamometer^™^); los valores mayores de 32 hombres y 20 kg en mujeres, se consideraron normales y se cl asificaron como saludables (26).
	Pruebas cortas de ejecución física (tres): equilibrio (asumir y mantener tres posiciones, pies juntos, semitándem y tándem; velocidad de la marcha (en 4 m), e incorporarse de una silla cinco veces (27). En cada prueba se puede obtener un máximo de 4 puntos. Las personas con 10 puntos o más se consideraron saludables.
Función cognitiva	Prueba cognitiva de Leganés. Los puntajes van de 0 a 32 puntos; los altos significan una adecuada función cognitiva. Se consideró normal un puntaje de >22 (20)
Bienestar psicológico	Escala de depresión del Centro de Estudios Epidemiológicos (CES-D), que consta de 20 ítems. Los puntajes totales están entre 0 y 60. Las personas con puntajes de <16 se consideraron sin depresión (28).
Bienestar social	Apoyo social: tener un confidente (29)
	Participación social en actividades en el hogar, comunitarias y religiosas (29)

### Modelos de envejecimiento saludable

Con base en los criterios previamente establecidos se construyeron tres modelos de envejecimiento saludable: el modelo multidimensional con las cinco dimensiones descritas; el modelo biomédico con tres dimensiones: salud fisiológica y metabólica, función física y función cognitiva, y, por último, el modelo psicosocial con las dimensiones de función cognitiva, bienestar psicológico y bienestar social.

### Variables independientes

Se registraron las variables de edad, sexo, nivel educativo y estado civil. Los ingresos se calcularon en salarios mínimos mensuales vigentes (SMLV) y se agruparon en tres categorías: pobre (ninguno o menos de un SMLV), medio (un SMLV) y alto (dos o más SMLV); la satisfacción con los ingresos se evaluó mediante la pregunta: "¿Usted considera sus ingresos...?", con tres opciones de respuesta: suficientes, aceptables o insuficientes ^19^. Se registró con quién vivía la persona en el momento de la entrevista y las opciones fueron: solo, con el cónyuge o con otros. La autopercepción de la salud se evaluó con la pregunta: "¿Usted considera su salud: buena, muy buena, regular, mala o muy mala?", cuya respuesta se clasificó como buena o muy buena, o como regular, mala o muy mala ^30^.

La presencia de comorbilidades se evaluó con base en el reporte de los propios participantes, mediante una lista de chequeo con las siguientes enfermedades crónicas: cáncer, enfermedad pulmonar, diabetes, hipertensión arterial, enfermedades del corazón, accidente o enfermedad cerebrovascular, osteoartrosis o artritis y osteoporosis. Para la composición corporal, se calculó el índice de masa corporal (IMC) en kg/m^2^. También, se indagó sobre los hábitos de tabaquismo y consumo de licor, y se registró si el participante fumaba o consumía licor, al menos, dos veces por semana en el momento de la encuesta.

### Análisis estadístico

Los modelos de envejecimiento saludable se construyeron con los criterios previamente establecidos. Se calculó la prevalencia de cada uno, se analizó su asociación con las covariables y se describió la prevalencia de cada uno de los modelos.

En el análisis del fenotipo de envejecimiento saludable, las variables se organizaron en dicotomías como se indica en el cuadro 1. Los porcentajes se compararon con la prueba de ji al cuadrado y la asociación se estableció con la razón de momios *(odds ratio,* OR). En todas las pruebas estadísticas se empleó un intervalo de confianza (IC) del 95 % y la significación estadística se estableció como p<0,05. Se calculó la prevalencia para cada uno de los modelos propuestos y la bondad del ajuste del modelo multidimensional se estableció mediante la prueba de Hosmer y Lemeshow. Para el procesamiento y análisis de la información, se utilizó el programa SPSS™, versión 24.0, para Windows.

### Consideraciones éticas

Se solicitó a los participantes el diligenciamiento de un consentimiento informado. El estudio fue aprobado por el Comité de Ética de la Facultad de Ciencias para la Salud de la Universidad de Caldas.

## Resultados

La muestra total fue de 407 personas, 50,1 % de las cuales eran mujeres con un promedio de edad de 69,2 años, sin diferencias estadísticas, lo cual respondía a los criterios de inclusión. En el cuadro 2, se presentan las características sociodemográficas y de salud de la población. En cuanto al estado civil, hubo mayor proporción de casados (49,6 %), seguidos por viudos (23,8 %), con diferencias estadísticas significativas por sexo (p=0,000), y un mayor número de hombres casados (33,6 %) y de mujeres viudas (20,1 %).

**Cuadro 2:** Características sociodemográficas y de salud de la población

	Hombres		Mujeres		Total	
Variables	n	%	n	%	n	%
	203	49,9	204	50,1	407	100
Edad (años)						
64-69	114	56,2	103	50,5	217	53,3
70-75	89	43,8	101	49,5	190	46,7
Estado civil						
Soltero	22	18,5	33	16,2	55	13,5
Casado	137	67,5	65	31,9	202	49,6
Viudo	15	7,4	82	40,2	97	23,8
Separado	29	14,3	24	11,8	53	13,0
Nivel educativo						
Primaria	157	77,3	183	89,7	340	83,5
Secundaria	10	4,9	10	4,9	20	4,9
Universitario**	36	17,7	11	5,4	47	11,5
Convivientes						
Solo	28	13,8	25	12,3	53	13,0
Cónyuge*	46	22,7	27	13,2	73	17,9
Otros	129	63,5	152	74,5	281	69,0
Ingresos económicos**						
Ninguno	41	20,2	70	34,3	111	27,3
<1 SMLV	45	22,2	59	28,9	104	25,6
1 SMLV	60	29,6	58	28,4	118	29,0
2-3 SMLV	38	18,7	15	7,4	53	13,0
4-5 SMLV	6	3,0	2	1,0	8	2,0
>5 SMLV	13	6,4	0	0	13	3,2
Satisfacción con los ingresos						
Suficientes	12	6,0	8	4,0	20	5,0
Aceptables	49	24,6	48	24,2	97	24,4
Insuficientes	138	69,3	142	71,7	280	70,5
Autopercepción de salud						
Muy buena o buena	112	55,1	93	45,8	205	63,1
Regular	78	38,4	98	48,3	176	43,3
Mala o muy mala	13	6,4	12	5,9	25	6,1
Número de enfermedades*						
0	67	33,0	28	13,8	95	23,3
1	64	31,5	64	31,5	128	31,5
2	47	23,2	54	26,6	101	24,9
3	18	8,9	39	19,2	57	14,0
4	4	2,0	12	5,9	16	3,9
5	3	1,5	3	1,5	6	1,5
6	0	0	2	1,0	2	0,5
7	0	0	1	0,5	1	0,2
IMC** (kg/m2)						
Bajo peso (<18,5)	10	4,9	6	2,9	16	3,9
Normal (18,5-24,9)	85	41,9	61	29,9	146	35,9
Sobrepeso (25-29,9)	89	43,8	89	43,6	178	43,7
Obesidad (≥30)	19	9,4	48	23,5	67	16,5
Tabaquismo (sí)	39	19,4	17	8,4	56	13,8
Alcohol (sí)**	24	11,9	1	0,5	27	6,6

SMLV: salario mínimo legal vigente. IMC: índice de masa corporal

* p<0,05

**p<0,001

En lo relacionado con el nivel educativo, la gran mayoría de participantes tenía estudios de básica primaria (83,5 %), el 4,9 % había cursado la secundaria, sin diferencias entre sexos, y el 11,5 % tenía estudios superiores, con una diferencia estadísticamente significativa entre sexos (p=0,000).

Casi las dos terceras partes de los participantes vivían con otras personas (69 %) y el 13 % vivía solo. El promedio de hombres que vivían con su cónyuge fue mayor (22,7 % hombres *Vs.* 13,2 % mujeres) (p=0,030).

En lo que respecta a los ingresos, casi la tercera parte no tenía ningún tipo de ingreso, en mayor proporción las mujeres (34,3 %). Entre una tercera y una cuarta parte recibía menos de uno o un salario mínimo mensual legal vigente.

Los hombres recibían mayores ingresos que las mujeres, con una diferencia significativa (p=0,000). En cuanto a la satisfacción con los ingresos, el 70,5 % los calificó como insuficientes, sin diferencias entre sexos.

Más de la mitad de las personas calificó su salud como buena o muy buena; hubo una mayor proporción de mujeres que la consideró regular, sin diferencias significativas entre sexos. Con respecto al número de comorbilidades, el 23,3 % refirió no tener ninguna enfermedad crónica. Más de la mitad (56,4 %) tenía entre una y dos enfermedades crónicas, y el 6,1 % reportó tener cuatro o más, con una diferencia estadísticamente significativa entre sexos (p=0,000). Así, el 33 % de los hombres y el 23,4 % de las mujeres manifestaron no tener ninguna de las enfermedades crónicas incluidas en la lista de chequeo y, el 35,5 % de los hombres y el 55 % de las mujeres, dos o más (p=0,001).

Considerando la clasificación de obesidad de la OMS basada en el IMC, el 3,9 % tenía bajo peso y el 16,5 % estaba en el rango de obesidad. Casi la mitad de los participantes tenía sobrepeso (43,7 %) y solamente el 35,9 % tenía un IMC normal, con diferencias estadísticamente significativas entre sexos (p=0,001).

El 19,4 % de las personas refirió tabaquismo, la mayoría hombres. En cuanto al licor, el 11,9 % de los hombres y el 0,5 % de las mujeres solían consumirlo en el momento de la entrevista, con diferencias estadísticamente significativas entre sexos (p=0,001).

El análisis discriminado de cada una de las dimensiones del fenotipo de envejecimiento saludable, se presenta en el cuadro 3.

**Cuadro 3:** Distribución de la población según las dimensiones del fenotipo de envejecimiento saludable

Dimensión	Criterio	Hombres		Mujeres		Total	
		n	%	n	%	n	%
Salud fisiológica y metabólica	Proteína C reactiva <1 mg/L	54	26,6	34	16,7	88	21,6
	Hemoglobina ‘glicosilada’ (HbA1c) <5,7 %	60	29,6	37	18,1	97	23,8
Función física	Fuerza de agarre (kg) >32 hombres y >20 mujeres	91	44,8	115	56,4	206	53,9
	Pruebas cortas de ejecución física 10-12 puntos*	134	66,0	110	53,9	244	60,0
Función cognitiva	Prueba cognitiva de Leganés>22 puntos	186	91,6	190	93,1	376	92,4
Bienestar psicológico	CES-D<16 puntos*	166	81,6	145	71,1	301	74,3
Bienestar social	Soporte social						
	Confidente (sí)*	155	76,4	175	85,5	330	81,1
	Participación en actividades						
	comunitarias (sí)**	41	17,8	113	55,4	154	37,8
	del hogar (sí)**	159	78,3	177	86,8	336	82,6
	religiosas (sí)**	179	88,2	192	94,1	371	91,2

* p<0,05 **p<0,001

En la dimensión de salud fisiológica y metabólica, se halló que solamente el 21,6 % registró un grado de inflamación medida con PCR por debajo de 1 mg/L; una proporción similar (23,8 %) registró un valor de <5,7 % en la prueba de hemoglobina 'glicosilada', con diferencias estadísticamente significativas entre sexos para ambos biomarcadores.

En la dimensión de la función física, un poco más de la mitad tenía una fuerza de agarre normal. En las pruebas cortas de ejecución física, el 60 % obtuvo entre 10 y 12 puntos, con diferencias entre sexos (66 % de los hombres *Vs.* 53,9 % de las mujeres) (p=0,013).

En la dimensión de la función cognitiva, la mayoría (92,4 %) de los participantes obtuvo 23 puntos o más en la prueba cognitiva de Leganés, sin diferencias entre hombres y mujeres. En cuanto a la dimensión de bienestar psicológico, el 74,3 % no registraba depresión según la escala CES-D *(Center for Epidemiologic Studies Depression Scale).*

Por último, en la dimensión de bienestar social, la gran mayoría de los participantes (81,1 %) contaba con un confidente, con diferencias entre los sexos (p=0,010). La participación en actividades religiosas y del hogar era alta, en tanto que solamente una tercera parte participaba en actividades comunitarias. Tanto el apoyo como la participación fueron más frecuentes en mujeres, con diferencias estadísticamente significativas (p=0,000).

**Figura 1:**
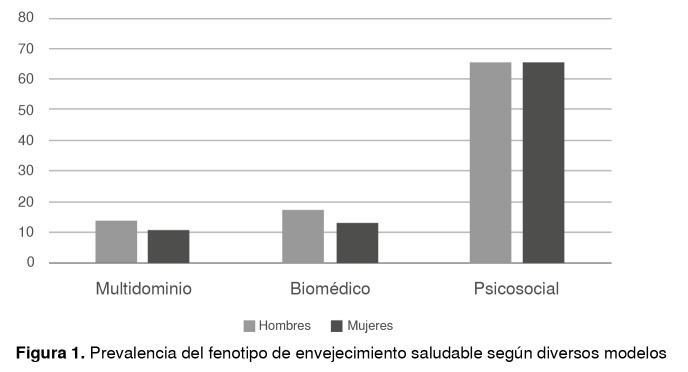
Prevalência del fenotipo de envejecimiento saludable según diversos modelos

La prevalência del fenotipo de envejecimiento saludable varió según el modelo analizado: 12,3 % en el modelo multidimensional, 15,5 % en el biomédico y aumentó a 66,3 % en el modelo psicosocial (figura 1). En el modelo multidimensional, la prevalência de envejecimiento saludable fue de 13,8 % en los hombres y de 10,8 % en las mujeres; en el modelo biomédico, de 17,2 % en los hombres y 13,7 % en las mujeres, y en el psicosocial, de 65,5 % en los hombres y de 67,2 % en las mujeres, sin diferencias estadísticas por sexo en ninguno de los modelos.

Los factores de predicción independientes fueron el tener buena o muy buena salud según la autopercepción y la satisfacción con los ingresos económicos. En los tres modelos, la autopercepción de una buena o muy buena salud fue el principal factor de predicción de envejecimiento saludable: en el modelo biomédico, se encontró una OR de 1,89 (IC_95%_ 1,05-3,40), en el multidimensional, de 2,13 (IC_950/_ 1,10-4,16) y, en el psicosocial, de 3,77 (IC_950/_ 2,35-6,05) (cuadro 4).

**Cuadro 4:** Factores de predicción del envejecimiento saludable

**Variable**	Modelo multidimensional		Modelo biomédico		Modelo psicosocial	
	OR	IC_95%_	OR	IC_95%_	OR	IC_95%_
Buena o muy buena salud, por autopercepción	2,13	1,10-4,16	1,89	1,05-3,40	3,77	2,35-6,05
Satisfacción con los ingresos	1,02	0,77-136	1,63	0,99-2,97	1,84	1,14-2,95
Estar casado	0,97	0,73-1,29	1,13	0,87-1,45	1,63	1,04-2,55
Sexo	1,10	0,73-1,64	1,13	0,82-1,55	1,11	0,77-1,59
Nivel educativo	1,00	0,95-1,05	1,2	0,88-1,62	1,03	0,97-1,10

OR: *Odds ratio*

La satisfacción con los ingresos registró una OR de 1,63 (IC_95%_ 0,99-2,97) en el modelo biomédico y una de 1,84 (IC_95%_ 1,14-2,95) en el modelo psicosocial.

En el análisis de regresión logística, esta variable despareció en el modelo multidimensional, aunque en el análisis bivariado la asociación fue significativa.

En el modelo psicosocial, un tercer factor asociado fue el estar casado, con una OR de 1,63 (IC_95_% 1,04-2,55), sin embargo, en los demás modelos su relación no fue significativa.

## Discusión

En este estudio, se presentan los primeros resultados sobre la prevalencia del fenotipo de envejecimiento saludable en personas colombianas mayores: 12,3 % según el modelo multidimensional, con cinco dimensiones (salud fisiológica y metabólica, función física, función cognitiva, función psicológica y función social); 15,5 % según un modelo biomédico de tres dimensiones (salud fisiológica y metabólica, función física y función cognitiva), y 66,3 % según el modelo psicosocial con sus dimensiones de función cognitiva, función psicológica y función social. En los tres modelos, los factores de predicción independientes para el envejecimiento saludable fueron tener buena o muy buena salud según la autopercepción y estar satisfechos con los ingresos económicos. En el modelo psicosocial, el estar casado también fue un factor de predicción.

La comparación con otros estudios es compleja dada la naturaleza arbitraria de la definición, las poblaciones incluidas, las dimensiones seleccionadas, los constructos y los puntos de corte, lo que da lugar a una considerable variación en la proporción de la población considerada saludable. En pocos estudios se ha intentado poner en funcionamiento el concepto de fototipo de envejecimiento saludable, utilizando enfoques integrales y multidimensionales, como el propuesto por Lara, *et al.*^18^ there is no single, simple and reliable measure of how healthily someone is ageing. Intervention studies need a panel of measures which capture key features of healthy ageing. To help guide our research in this area, we have adopted the concept of the \"Healthy Ageing Phenotype\" (HAP, que sirvió de base para el presente estudio.

Los estudios actuales se han basado en medidas parciales o indirectas de cada uno de los dominios asociados con dicho fenotipo. Los índices disponibles se enfocan en diferentes combinaciones de dominios, pero comúnmente lo hacen en la función cognitiva y la capacidad física ^15^.

Como ya se mencionó, este fenotipo se basa en el envejecimiento exitoso, el cual diferencia entre los individuos con enfermedades y aquellos con discapacidades ^6^^,^^31^. En algunos estudios, los puntos de corte de las mediciones seleccionadas se basan en la distribución de la población de estudio y se ajustan para capturar aquellos que se ubican por encima de la mediana ^32^, en el tercil superior ^33^, el cuartil ^34^ o el quintil ^35^.

Debido a estas diferencias, las prevalencias de envejecimiento saludable son muy variables, entre el 4,5 y el 80 %, dependiendo de los aspectos incluidos ^21^^,^^36^^-^^38^. Más allá de las dificultades para su definición y la variedad de las prevalencias, se han señalado otros aspectos problemáticos, como cuáles de estas variables son resultado del envejecimiento saludable, cuáles son constitutivas y cuáles operan como factores de predicción ^37^, por lo que aún se requiere mucha investigación para lograr un consenso conceptual que precise estos aspectos.

Por otra parte, las diferencias de las prevalencias en los tres modelos se deben a las dimensiones incluidas. El modelo multidimensional aporta una evaluación más integral al considerar cinco dimensiones, incluidos los aspectos fisiológicos y metabólicos, que no se tienen en cuenta en los otros modelos. La prevalencia de envejecimiento saludable según este modelo, fue la más baja en el presente estudio (12,3 %), lo que se explica por la gran proporción de personas con alteraciones en el metabolismo de la glucosa (prueba de hemoglobina 'glicosilada') y con procesos inflamatorios (PCR). Se ha establecido que la inflamación crónica está estrechamente asociada con la mayoría de las enfermedades crónicas relacionadas con la edad y con factores de riesgo cardiovascular ^39^, sarcopenia, obesidad y diabetes ^40^, así como con el deterioro funcional y la discapacidad ^24^.

Sin embargo, al eliminar las dimensiones de función psicológica y social, la prevalencia del envejecimiento saludable en el modelo biomédico se incrementa en dos puntos porcentuales; no obstante, si se consideran solamente los aspectos psicosociales, esta aumenta hasta 63,3 %, lo cual obliga a preguntarse cuáles elementos tienen más peso en el proceso de envejecimiento más allá de los aspectos biomédicos, y refuerza la necesidad de reconocer que el envejecimiento saludable no es simplemente lo opuesto al envejecimiento con enfermedad o deterioro funcional ^7^^,^^18^^,^^38^.

La autopercepción de tener buena salud fue un factor de predicción del fenotipo de envejecimiento saludable en los tres modelos analizados. La autopercepción de la salud se acepta ampliamente como una medida sensible y confiable del estado de salud general ^41^; en ella participan la función física ^42^, la presencia de enfermedades, y la existencia de discapacidades ^43^ y limitaciones funcionales y cognitivas ^44^. En muchos estudios se sugiere que también está determinada por otros aspectos, como el apoyo social y la religiosidad ^45^. Por ello, no sorprende la relación entre el envejecimiento saludable y la autopercepción de buena salud.

En este estudio, cerca de la mitad de los participantes consideraba que su salud era muy buena o buena, proporción similar a la hallada en otros estudios ^46^. Estos porcentajes varían entre estudios, pero no se han modificado sustancialmente en los últimos años; por lo general, se informa que entre una tercera parte y la mitad de la población anciana se considera saludable ^44^^,^^46^.

En Colombia, hay pocos estudios sobre la autopercepción de los ancianos de su estado de salud. En el de Gómez, *et al.*^47^, se reportó que la mitad de la población anciana que vive en la comunidad de Manizales se consideraba saludable, porcentaje similar al de este estudio. Los investigadores reportaron una asociación importante con enfermedades crónicas, problemas de visión y capacidad funcional, especialmente en el ámbito físico de las actividades de la vida diaria. En un estudio realizado en Cali, el 60 % de los participantes reportó buena salud ^48^ y, en el seguimiento un año después, se encontró que aquellos con una autopercepción de mala salud presentaban un mayor deterioro y aparición de síndromes geriátricos, así como una mayor frecuencia en el uso de servicios de salud ^49^. En la *Encuesta* Nacional de Salud, Bienestar y Envejecimiento, SABE, en Colombia ^50^, se reportó la misma proporción de autopercepción de buena o muy buena salud (51,3 %), siendo mayor en los estratos altos, en zonas urbanas y en personas con mayor nivel educativo.

El ingreso económico es una variable que se asocia constantemente con el fenotipo de envejecimiento saludable ^51^, lo que refuerza la importancia de los factores sociales determinantes de la salud y de la inequidad y sus consecuencias en la salud, como ha sido ampliamente documentado en la literatura especializada ^52^. Por ejemplo, la proporción de adultos mayores que se clasifican como 'de alto funcionamiento' es sustancialmente mayor entre las personas con un nivel elevado de ingresos ^53^ y muy poco se asocian las dificultades económicas con la buena salud ^54^. El papel del ingreso como mediador de la salud no se ha explorado de forma exhaustiva, más que como un simple factor de predicción del envejecimiento saludable. Las personas con mayores niveles de educación suelen tener empleos mejor pagados y, en consecuencia, mayores recursos financieros; esto puede aumentar las probabilidades de un envejecimiento saludable al permitirles acceder a una mejor atención médica o residir en mejores vecindarios ^55^.

Por otra parte, los resultados de este estudio evidenciaron que, según el modelo psicosocial, el estar casado es un factor independiente de predicción del envejecimiento saludable. El estado marital también se ha asociado con envejecer bien ^56^. Estos hallazgos refuerzan el concepto del papel protector del matrimonio en la salud de las personas mayores y son varias las explicaciones que se han propuesto: el apoyo social y económico, la selección de los individuos más sanos para casarse y permanecer casado, mejores conductas y hábitos de salud entre los casados y, por último, el estrés producido por el duelo de la disolución marital ^57^.

El fenotipo de envejecimiento saludable ofrece una evaluación positiva e integral de las personas mayores. Este estudio podría servir de base para ulteriores análisis que incluyan otros rangos de edad, diseños longitudinales y comparaciones entre poblaciones de diferentes regiones, lo que ayudaría a ahondar en el conocimiento en esta área y a esclarecer aspectos como la alta prevalencia de envejecimiento saludable observada en el modelo psicosocial, lo cual es de gran relevancia para algunos investigadores a pesar de la presencia de comorbilidades o de algún grado de compromiso funcional ^58^.

Las fortalezas del estudio son varias: hasta donde se pudo establecer, es el primero en el que se estima la prevalencia de envejecimiento saludable en personas mayores colombianas desde la perspectiva de tres modelos diferentes. La determinación de los factores relacionados con su presencia permite establecer un perfil de personas mayores saludables, con buen funcionamiento mental, emocional y físico, y decididamente, comprometidos con la vida, que mantienen sus relaciones interpersonales y participan en actividades significativas en sus comunidades.

Entre las limitaciones del estudio, se destaca su diseño trasversal, que no permite establecer relaciones de causa y efecto entre las variables. Otra limitación fue el rango de edad de la muestra estudiada, de 65 a 74 años, lo que impide generalizar los resultados a adultos mayores de 75 años.

La determinación de la prevalencia, los indicadores y los factores asociados con el envejecimiento saludable, posibilita su empleo en la evaluación y el análisis de esta población en la atención primaria en salud a partir de una visión multidimensional. Asimismo, permite establecer indicadores claros de evaluación del impacto de las actividades propuestas en las políticas públicas orientadas a este grupo poblacional. Por ello, es esencial que los equipos de salud pública se apropien de este nuevo paradigma y del enfoque multidimensional.

En conclusión, la prevalencia de envejecimiento saludable fue baja en los modelos biológico y multidimensional (1 de cada 10), y mayor, en el modelo psicosocial (6 de cada 10). A pesar de ello, los factores independientes de predicción fueron los mismos: la autopercepción de tener una buena o muy buena salud, la satisfacción con los ingresos económicos y el estar casado.
